# Cognitive–Motor Coupling in Multiple Sclerosis: Do Chronological Age and Physical Activity Matter?

**DOI:** 10.3390/brainsci15030274

**Published:** 2025-03-05

**Authors:** Brenda Jeng, Peixuan Zheng, Robert W. Motl

**Affiliations:** Department of Kinesiology and Nutrition, University of Illinois Chicago, Chicago, IL 60612, USA; bjeng@uic.edu (B.J.); pxzheng@uic.edu (P.Z.)

**Keywords:** cognition, physical function, neurorehabilitation, aging, physical activity

## Abstract

**Background**: People with multiple sclerosis (MS) often demonstrate both cognitive and physical dysfunctions, particularly with greater age and lower physical activity levels, and there is evidence of a relationship between these outcomes (i.e., cognitive–motor coupling) in MS. To date, little is known about cognitive–motor coupling when controlling for chronological age and levels of physical activity. **Objectives**: We examined cognitive–motor coupling in people with MS while accounting for chronological age and physical activity. **Methods**: The sample included 290 people with MS between the ages of 22 and 77 years. Participants underwent the Symbol Digit Modalities Test (SDMT) for cognitive processing speed and the California Verbal Learning and Memory Test–Second Edition (CVLT-II) for verbal learning and memory. Participants completed the 6-Minute Walk and the Timed 25-Foot Walk tests for walking endurance and speed, respectively. Participants wore an accelerometer for a 7-day period to measure moderate-to-vigorous physical activity (MVPA). **Results**: The bivariate correlation analyses indicated that cognitive function had moderate-to-strong associations with motor function (range of *r_s_* between 0.433 and 0.459). The linear regression analyses indicated cognitive–motor coupling between SDMT and motor function (with a range of β between 0.139 and 0.145) when controlling for demographic and clinical characteristics. The regression analyses further indicated that the CVLT-II was associated with motor function (with a range of β between 0.125 and 0.135) when controlling for demographic and clinical characteristics. When age and MVPA were entered into the regression analyses, SDMT was still associated with the motor function of individuals (β = 0.119), and CVLT-II was still associated with the motor function of individuals (with a range of β between 0.115 and 0.124). **Conclusions:** Cognitive–motor coupling is present in people with MS independent of chronological age and levels of physical activity. This warrants further investigation of the underlying mechanism and potential approaches for the management of co-occurring MS-related dysfunction.

## 1. Introduction

Cognitive and walking dysfunctions are prevalent, disabling, and often co-occurring for inter-related consequences of multiple sclerosis (MS) [1]. The relationship between cognitive and motor functions in MS has been termed cognitive–motor coupling [2]. The presence of cognitive–motor coupling was first reported in a study of people with MS and controls wherein cognitive processing speed, based on the Symbol Digit Modalities Test (SDMT), was independently associated with walking speed, based on the Timed 25-Foot Walk (T25FW) test, and the association was stronger in MS than controls [2]. Other researchers have reported cognitive–motor coupling based on associations between SDMT and T25FW as well as the 6-Minute Walk test (6MW; walking endurance) performance in people with MS [3]. This strong relationship may suggest that both cognitive and motor functions involve the particular overlap of brain regions subserving these functions in MS [4,5,6] that might further be associated with age and/or physical activity. There is value in understanding cognitive–motor coupling [7,8] as possibly an age-related issue and identifying physical activity as a potential modifiable behavior with value in mitigating this phenomenon.

To date, the studies that have examined cognitive–motor coupling in MS have primarily involved young and middle-aged samples, yet there is a demographic shift such that MS is present across the entire lifespan, particularly in older adults [9]. Some research has focused on cognitive–motor coupling in older adults with MS and across the lifespan of individuals. For example, one study recruited a sample of older adults with MS and controls matched by age and sex and reported moderate-to-strong relationships between SDMT and T25FW as well as 6MW in MS (i.e., cognitive–motor coupling) but no relationships in controls [10]. Another study examined physical and cognitive function across the lifespan in MS and controls and identified moderate-to-strong relationships in MS but weak relationships in controls [10]. This study further detected strong relationships between cognitive and motor performance in young and middle-aged samples with MS [11].

To date, there has been a dearth of research examining physical activity as an underlying factor for explaining cognitive–motor coupling in MS, despite a conceptual paper that argues for such an inquiry based on proposed synergistic effects of physical activity on cognitive and motor outcomes [12]. This is intriguing, as the first study on cognitive–motor coupling in MS identified physical activity and exercise behavior as possible explanations for the association between variables based on the putative effects on underlying brain parameters [2]. To that end, cardiorespiratory fitness, a physiological surrogate of participating in regular aerobic physical activity, accounted for the association between cognitive and motor outcomes [13,14], whereas deep grey matter brain structures (e.g., thalamus) did not in a small sample of young and middle-aged people with MS [13]. Importantly, cardiorespiratory fitness is a characteristic that reflects the contributions of physical activity behavior in individuals, but it further reflects genetic and other lifestyle variables and is not directly amenable to modifications through interventions, unlike physical activity behavior [15,16].

The current study examined cognitive–motor coupling in people with MS while accounting for chronological age and physical activity. Chronological age might represent a confounding variable, as both walking and cognitive function decline with increasing age in MS [11]. Physical activity might account for cognitive–motor coupling based on conceptual arguments [2,12] and recent evidence linking cardiorespiratory fitness with the association between cognitive and motor outcomes in MS [13,14].

## 2. Materials and Methods

### 2.1. Participants

This cross-sectional study involved a secondary analysis of data from 5 studies [10,17,18,19,20]. Participants with MS in those studies were included based on the following criteria: (1) MS diagnosis; (2) relapse-free in the last 30 days; (3) an age of at least 18 years; (4) ability to walk with or without an assistive device; and (5) willingness to complete the assessments.

### 2.2. Outcome Measures

In the aforementioned studies, demographic information was collected using a laboratory-standardized questionnaire, including age, sex, years of education, and race. Race was dichotomized into Caucasian and non-Caucasian categories. Participants with MS further provided clinical information, including disease duration. Ambulatory disability status was assessed using the one-item Patient-Determined Disease Steps (PDDS) scale [21]. PDDS scale scores have been strongly correlated with scores from the Expanded Disability Status Scale [21], and therefore, the PDDS has been identified as a reasonable surrogate for capturing disability status in MS [22].

The SDMT and CVLT-II assessed cognitive processing speed and verbal learning and memory, respectively. For the SDMT, participants were presented with a series of symbols and instructed to verbally indicate the digit that was paired with each symbol according to the key located at the top of the page [23]. Participants were instructed to work as quickly as possible, and we recorded the total correct responses in 90 s. For the CVLT-II, the examiner read a list of 16 words aloud, and participants were instructed to recall as many of the words as possible, in any order, and this process was repeated for another 4 trials [24]. We recorded the total correct responses from the five trials for a sum. 

The 6MW and T25FW assessed walking endurance and speed, respectively. For the 6MW, participants were instructed to walk as far and as fast as possible within the limits of their stability in a 6-min period [25]. We recorded the total distance covered in meters. For the T25FW, participants were instructed to walk a marked 25-foot course as quickly and safely as possible [26]. We recorded the time to complete each of the two trials and averaged the time of the two trials to calculate speed.

Free-living physical activity was measured using an ActiGraph GT3X+ accelerometer (ActiGraph LLC, Pensacola, FL, USA). We placed the accelerometer in a pouch on a belt, and participants wore the belt around their waist with the accelerometer placed above the non-dominant hip. Participants further wore the device for 7 days during waking hours except during water-based activities (i.e., showering, swimming) and recorded periods of wear time in a daily log; this was inspected to verify the wear time during data processing. The data were downloaded and processed into one-minute epochs with low-frequency extension in ActiLife, version 6 [27]. Data were included based on a daily total wear time of at least 600 min (i.e., 10 h); participants with at least one valid day of data were included in the analyses. The outcome of interest was time spent undertaking moderate-to-vigorous physical activity (MVPA; minutes/day).

### 2.3. Procedures

The studies were conducted in accordance with the Declaration of Helsinki, and the procedures of the 5 studies were approved by appropriate Institutional Review Boards. All participants provided written consent prior to data collection. Participants provided demographic and clinical information and then completed assessments of cognitive and motor function. We further provided participants with an accelerometer on a belt, a daily log, and a pre-stamped, pre-addressed envelope for return service via the United States Postal Service at the end of the visit.

### 2.4. Data Analysis

The data were analyzed using SPSS, version 29 (SPSS, IBM Statistics, Armonk, NY, USA). We provided descriptive statistics for demographic, clinical, and physical activity variables, as well as cognitive and motor assessments. We then examined associations among cognitive (SDMT, CVLT-II) and motor (6MW, T25FW) outcomes, along with the demographic, clinical, and physical activity variables, using Spearman’s rank-order bivariate correlations [28]. We finally performed a series of stepwise, linear regression analyses. We regressed motor function (6MW or T25FW) based on demographic characteristics (sex, years of education, and race) in Step 1, clinical characteristics (disease duration and PDDS) in Step 2, cognitive function (SDMT or CVLT-II) in Step 3 (i.e., cognitive–motor coupling), and age and physical activity in Step 4 (i.e., independence of cognitive–motor coupling).

## 3. Results

We provide descriptive statistics of participant characteristics in Table 1. The mean age of participants was 53.0 (13.3) years, and the sample consisted mainly of females (76%) and Caucasians (65%). Participants had a mean disease duration of 14.6 (9.6) years and a median PDDS score of two (IQR of three). On average, the sample engaged in 19.9 (20.2) min/day of MVPA.

The descriptive statistics for outcomes of cognitive and motor function are presented in Table 2. The sample spanned greater ranges in age, education level, race, and disease duration but had a similar ratio of females to males, MS type, and disability level, compared with the initial study on cognitive–motor coupling in MS [2].

The bivariate correlations among outcomes of cognitive function, motor function, age, physical activity, and demographic and clinical characteristics are presented in Table 3. The results indicate that SDMT scores had moderate-to-strong associations with 6MW (*r_s_* = 0.459) and T25FW (*r_s_* = 0.433), whereas CVLT-II scores had moderate associations with 6MW (*r_s_* = 0.370) and T25FW (*r_s_* = 0.322). Of note, age and MVPA were both associated with cognitive and motor outcomes, supporting the linear regression analyses examining cognitive–motor coupling while accounting for chronological age and physical activity.

The linear regression analyses are provided in Table 4, Table 5, Table 6 and Table 7. The linear regression analyses indicate cognitive–motor coupling between SDMT and 6MW (β = 0.139, *p* = 0.003) and SDMT and T25FW (β = 0.145, *p* = 0.010) when controlling for sex, years of education, race, disease duration, and disability status (Table 4 and Table 5). Moreover, CVLT-II scores were associated with both 6MW (β = 0.135, *p* = 0.004) and T25FW (β = 0.125, *p* = 0.019) when controlling for demographic and clinical characteristics (Table 6 and Table 7). When age and MVPA were entered into the regression analyses, the associations remained significant between SDMT with 6MW and T25FW (β = 0.119 and 0.119, *p* = 0.012 and 0.036, respectively), as well as between CVLT-II with 6MW and T25FW (β = 0.124 and 0.115, *p* = 0.006 and 0.029, respectively).

## 4. Discussion

This cross-sectional study examined cognitive–motor coupling while accounting for chronological age and physical activity in people with MS. Our bivariate correlation analyses indicated significant associations among cognitive and motor function outcomes. These relationships were further confirmed by the linear regression analyses, even after controlling for sex, years of education, race, disease duration, and disability status. Notably, the relationships among cognitive and motor function outcomes remained significant when accounting for age and MVPA in the linear regression models. These results suggest that (1) chronological age does not represent a confounding variable, despite its association with the decline in both physical and cognitive function in MS [11], and (2) physical activity does not account for cognitive–motor coupling, despite conceptual arguments [2,12] and recent evidence of cardiorespiratory fitness accounting for the association between cognitive and motor outcomes [13,14].

There is consistent evidence of cognitive–motor coupling based on bivariate correlations between performance scores for the measures of cognitive and motor function in people with MS. For example, multiple studies have reported moderate associations between cognitive processing speed and motor function outcomes in people with MS [2,3,10], and some studies have reported small-to-moderate or no association between verbal learning and memory and motor function outcomes [2,10]. Our bivariate correlation analyses further revealed significant relationships among cognitive and motor function outcomes in people with MS. This finding is notable, as it establishes cognitive–motor coupling in a manner consistent with previous research before examining the influences of age and physical activity. This replication of cognitive–motor coupling in MS is further important given recent and broad concerns regarding the reproducibility of results in science [29,30].

The presence of cognitive–motoring coupling has been established when controlling for a range of demographic and clinical variables in MS [2,10]. For example, the original paper on cognitive–motoring coupling in MS accounted for demographic (sex, years of education) and clinical (disease duration, disability status) characteristics [2]. We accounted for a similar set of variables as well as race when examining cognitive–motor coupling in MS, as race is an important covariate based on its association with cognitive and motor outcomes in MS [31]. We observed consistent associations among cognitive and motor outcomes even after controlling for demographic (sex, years of education, race) and clinical (disease duration, disability status) characteristics in the current study. This replication demonstrated the independence of cognitive–motor coupling from demographic and clinical variables in MS, and this is also critical given concerns regarding the reproducibility of the results in science [29].

Our results further indicate that cognitive–motor coupling remained even after accounting for age in people with MS. This finding is important, as increasing age has consistently been associated with worse cognitive and motor functions in MS [11,17], making age a potential confounder of cognitive–motor coupling. Some research studies have reported a significant relationship between cognitive and motor outcomes among older adults with MS [10] as well as young and middle-aged adults with MS [11]. Interestingly, we provide evidence that age is associated with cognitive and motor outcomes in this study, but this did not account for cognitive–motor coupling in the regression analyses. This suggests that the presence of cognitive–motor coupling independent of age and the worsening of cognitive and motor functions with increases in age in MS [11] does not explain the observed association between cognitive and motor outcomes.

The current study focused on physical activity and its influence on cognitive–motor coupling in people with MS. This is important, as physical activity has consistently been associated with better cognitive and motor functions in MS [17,32], and there are further conceptual arguments [2,12] that support such an examination [13,14]. Our results indicate that physical activity is associated with both cognitive and motor outcomes based on the bivariate analyses, but it does not account for cognitive–motor coupling in people with MS when included in the regression analyses. This seemingly runs counter with a recent report which states that cardiorespiratory fitness, a physiological surrogate of regular aerobic physical activity participation, accounts for the association between cognitive and motor outcomes [13,14], whereas deep grey matter brain structures (e.g., thalamus) do not, in a small sample of young and middle-aged people with MS [13]. Perhaps the results of that study reflected the small sample and/or the focus on a physiological characteristic of a person as a surrogate of physical activity, and our results with larger sample sizes and the direct measurement of physical activity might argue against the involvement of physical activity in cognitive–motor coupling in MS.

We acknowledge several limitations of our research. One limitation was the cross-sectional design of the study, as it does not permit inferences on causality. This limitation could be overcome in future research by examining longitudinal changes in cognitive–motor coupling as a function of disease progression. Another limitation was the lack of data on MS-related symptoms such as depression, as this has been associated with cognitive and motor performance in MS [33] and might account for cognitive–motor coupling. We did not examine the influence of comorbid conditions (i.e., cardiovascular disease, osteoarthritis) or medications, and these might independently or concurrently influence cognitive and motor function.

There are multiple avenues for future research on cognitive–motor coupling in MS. One potential avenue for future research may include examining the associations between cognitive [34] and physical [35] reserve, either separately or combined, with cognitive–motor coupling in MS. There is evidence that cognitive and physical reserve are associated with cognitive and motor outcomes and that people with MS who have both lower cognitive and physical reserves demonstrate significantly worse cognitive and motor outcomes [36]. This pattern suggests that having a higher cognitive or physical reserve, or both, may have protective effects on cognitive–motor coupling in people with MS. Another avenue for future research includes identifying magnetic resonance imaging metrics such as global brain atrophy or lesion burden and location as correlates of cognitive–motor coupling [4,37,38]. One other direction may include the development of screening tools that are valid for detecting cognitive–motor deficits, particularly in the early stages of the disease, where deficits may precede disease manifestations and become sensitive to changes over time [8,39,40]. Other research directions may explore modifiable approaches beyond physical activity for managing the relationship between cognitive and motor functions in this population, as deficits in cognitive–motor coupling can interfere with daily tasks such as talking while walking or driving [41,42]. One final research avenue might involve examining the relationship between cognitive–motor coupling and fall risk [43], as this might inform future fall prevention programs for researchers and clinicians [41].

## 5. Conclusions

Cognitive–motor coupling is consistently present in samples of people with MS, and we report here, for the first time, that this is independent of chronological age and physical activity as well as other demographic and clinical variables. This warrants further investigation of the underlying mechanism and potential approaches to managing co-occurring MS-related dysfunctions.

## Figures and Tables

**Table 1:** Demographic, clinical, and physical activity characteristics of the sample of people with multiple sclerosis.

Characteristics	Statistics
Age, *years*	53 (22–77)
Sex, *n*, *%*	
Female Male	221, 76% 69, 24%
Race	
Caucasian Non-Caucasian	190, 65% 100, 34%
Education, *years*	16 (9–21)
MS type, *n*, *%*	
Relapsing-remitting Progressive	253, 89% 31, 11%
Disease duration, *years*	15 (1–48)
PDDS, *0–8*	2 (0–7)
MVPA, *min/day*	20 (0–135)

Notes: Data are presented as mean (range), number, or percentage. MS, multiple sclerosis; PDDS, Patient-Determined Disease Steps; MVPA, moderate-to-vigorous physical activity.

**Table 2:** Descriptive statistics for outcomes of cognitive and motor function in the sample of people with multiple sclerosis.

Variables	Mean (SD)
SDMT, *score*	48.3 (12.5)
CVLT-II, *score*	44.6 (10.3)
6MW, *meters*	438.3 (140.1)
T25FW, *meters per sec*	1.6 (0.5)

Notes: SDMT, Symbol Digit Modalities Test; CVLT-II, California Verbal Learning Test–Second Edition; 6MW, 6-Minute Walk; T25FW, Timed 25-Foot Walk.

**Table 3:** Bivariate correlations among outcomes of cognitive function, motor function, age, physical activity, and demographic and clinical characteristics in the sample of people with multiple sclerosis.

	1	2	3	4	5	6	7	8	9	10
1. SDMT										
2. CVLT-II	0.490 **									
3. 6MW	0.459 **	0.370 **								
4. T25FW	0.433 **	0.322 **	0.911 **							
5. Age	−0.347 **	−0.161 *	−0.258 **	−0.316 **						
6. MVPA	0.316 **	0.218 *	0.665 **	0.617 **	−0.278 **					
7. Sex	−0.129 *	−0.170 **	0.090	0.072	0.017	0.216 **				
8. Education	0.388 **	0.339 **	0.237 **	0.150 *	−0.037	0.139 *	0.033			
9. Race	−0.085	−0.214 **	−0.171 **	−0.172 **	−0.374 **	−0.117	−0.129 *	−0.213 **		
10. Disease Duration	−0.237 **	−0.106	−0.220 **	−0.222 **	0.648 **	−0.274 **	−0.110	−0.069	−0.270 **	
11. PDDS	−0.401 **	−0.334 **	−0.742 **	−0.680 **	0.272 **	−0.487 **	0.001	−0.188 **	0.007	0.184 **

Notes: *p*-value of 0.05 *; *p*-value of <0.001 **. SDMT, Symbol Digit Modalities Test; CVLT-II, California Verbal Learning Test–Second Edition; 6MW, 6-Minute Walk; T25FW, Timed 25-Foot Walk; MVPA, moderate-to-vigorous physical activity; PDDS, Patient-Determined Disease Steps.

**Table 4:** Hierarchical linear regression analysis of Symbol Digit Modalities Test scores and 6-Minute Walk scores in the sample of people with multiple sclerosis.

	Predictor	*B*	*SE B*	β	*p*-Value
Step 1	Sex	154.668	68.208	0.139	0.024
	Years of Education	47.572	12.665	0.232	<0.001
	Race	−92.904	61.248	−0.095	0.131
Step 2	Sex	131.144	45.660	0.118	0.004
	Years of Education	20.934	8.491	0.102	0.014
	Race	−135.150	42.513	−0.138	0.002
	Disease Duration	−4.376	2.144	−0.088	0.042
	PDDS	−179.418	10.336	−0.710	<0.001
Step 3	Sex	149.109	45.359	0.134	0.001
	Years of Education	13.473	8.729	0.066	0.124
	Race	−114.629	42.422	−0.117	0.007
	Disease Duration	−3.035	2.158	−0.061	0.161
	PDDS	−167.509	10.936	−0.663	<0.001
	SDMT	5.386	1.811	0.139	0.003
Step 4	Sex	104.905	45.221	0.094	0.021
	Years of Education	14.109	8.476	0.069	0.097
	Race	−100.359	44.618	−0.102	0.025
	Disease Duration	−1.764	2.555	−0.035	0.491
	PDDS	−151.852	11.224	−0.601	<0.001
	SDMT	4.613	1.831	0.119	0.012
	Age	−0.596	2.043	−0.017	0.771
	MVPA	4.179	0.999	0.182	<0.001
*R*^2^ = 0.092 for Step 1; *R*^2^ = 0.611 for Step 2; *R*^2^ = 0.615 for Step 3; *R*^2^ = 0.638 for Step 4.					

Notes: PDDS, Patient-Determined Disease Steps; SDMT, Symbol Digit Modalities Test; 6MW, 6-Minute Walk; T25FW, MVPA, moderate-to-vigorous physical activity.

**Table 5:** Hierarchical linear regression analysis of Symbol Digit Modalities Test scores and Timed 25-Foot Walk scores in the sample of people with multiple sclerosis.

	Predictor	*B*	*SE B*	β	*p*-Value
Step 1	Sex	0.159	0.084	0.128	0.060
	Years of Education	0.035	0.015	0.153	0.025
	Race	−0.128	0.076	−0.116	0.091
Step 2	Sex	0.150	0.059	0.121	0.012
	Years of Education	0.007	0.011	0.031	0.522
	Race	−0.219	0.054	−0.198	<0.001
	Disease Duration	−0.007	0.003	−0.137	0.007
	PDDS	−0.198	0.014	−0.682	<0.001
Step 3	Sex	0.171	0.059	0.138	0.004
	Years of Education	−0.001	0.011	−0.005	0.925
	Race	−0.188	0.055	−0.170	<0.001
	Disease Duration	−0.006	0.003	−0.104	0.042
	PDDS	−0.184	0.015	−0.632	<0.001
	SDMT	0.006	0.002	0.145	0.010
Step 4	Sex	0.125	0.060	0.101	0.038
	Years of Education	0.000	0.011	−0.001	0.975
	Race	−0.189	0.059	−0.170	0.002
	Disease Duration	−0.002	0.003	−0.035	0.560
	PDDS	−0.166	0.015	−0.570	<0.001
	SDMT	0.005	0.002	0.119	0.036
	Age	−0.003	0.003	−0.088	0.189
	MVPA	0.004	0.001	0.154	0.003
*R*^2^ = 0.060 for Step 1; *R*^2^ = 0.557 for Step 2; *R*^2^ = 0.571 for Step 3; *R*^2^ = 0.593 for Step 4.					

Notes: PDDS, Patient-Determined Disease Steps; SDMT, Symbol Digit Modalities Test; T25FW, Timed 25-Foot Walk; MVPA, moderate-to-vigorous physical activity.

**Table 6:** Hierarchical linear regression analysis of California Verbal Learning Test–Second Edition scores and 6-Minute Walk scores in the sample of people with multiple sclerosis.

	Predictor	*B*	*SE B*	β	*p*-Value
Step 1	Sex	153.549	68.136	0.138	0.025
	Years of Education	47.374	12.651	0.231	<0.001
	Race	−89.223	61.008	−0.091	0.145
Step 2	Sex	129.612	45.625	0.116	0.005
	Years of Education	20.698	8.486	0.101	0.015
	Race	−133.087	42.452	−0.136	0.002
	Disease Duration	−4.527	2.137	−0.091	0.035
	PDDS	−179.307	10.333	−0.709	<0.001
Step 3	Sex	160.396	46.141	0.144	<0.001
	Years of Education	13.727	8.688	0.067	0.115
	Race	−102.691	43.072	−0.105	0.018
	Disease Duration	−3.437	2.138	−0.069	0.109
	PDDS	−170.341	10.625	−0.674	<0.001
	CVLT-II	6.187	2.103	0.135	0.004
Step 4	Sex	116.191	45.884	0.104	0.012
	Years of Education	13.777	8.406	0.067	0.102
	Race	−91.620	44.662	−0.094	0.041
	Disease Duration	−1.628	2.542	−0.033	0.523
	PDDS	−153.059	11.029	−0.605	<0.001
	CVLT-II	5.668	2.050	0.124	0.006
	Age	−1.185	1.969	−0.033	0.548
	MVPA	4.205	.996	0.183	<0.001
*R*^2^ = 0.090 for Step 1; *R*^2^ = 0.610 for Step 2; *R*^2^ = 0.623 for Step 3; *R*^2^ = 0.650 for Step 4.					

Notes: PDDS, Patient-Determined Disease Steps; CVLT-II, California Verbal Learning Test–Second Edition; 6MW, 6-Minute Walk; MVPA, moderate-to-vigorous physical activity.

**Table 7:** Hierarchical linear regression analysis of California Verbal Learning Test–Second Edition scores and Timed 25-Foot Walk scores in the sample of people with multiple sclerosis.

	Predictor	*B*	*SE B*	β	*p*-Value
Step 1	Sex	0.158	0.286	0.128	0.060
	Years of Education	0.035	0.084	0.153	0.025
	Race	−0.127	0.015	−0.115	0.094
Step 2	Sex	0.149	0.059	0.121	0.012
	Years of Education	0.007	0.011	0.030	0.529
	Race	−0.218	0.054	−0.198	<0.001
	Disease Duration	−0.007	0.003	−0.139	0.006
	PDDS	−0.198	0.014	−0.682	<0.001
Step 3	Sex	0.181	0.060	0.146	0.003
	Years of Education	−0.001	0.011	−0.004	0.931
	Race	−0.187	0.055	−0.170	<0.001
	Disease Duration	−0.006	0.003	−0.115	0.019
	PDDS	−0.190	0.014	−0.655	<0.001
	CVLT-II	0.006	0.003	0.125	0.019
Step 4	Sex	0.135	0.060	0.109	0.027
	Years of Education	−0.001	0.011	−0.006	0.905
	Race	−0.189	0.058	−0.172	0.001
	Disease Duration	−0.002	0.003	−0.035	0.561
	PDDS	−0.170	0.015	−0.583	<0.001
	CVLT-II	0.006	0.003	0.115	0.029
	Age	−0.004	0.003	−0.108	0.092
	MVPA	0.004	0.001	0.155	0.003
*R*^2^ = 0.060 for Step 1; *R*^2^ = 0.557 for Step 2; *R*^2^ = 0.569 for Step 3; *R*^2^ = 0.593 for Step 4.					

Notes: PDDS, Patient-Determined Disease Steps; CVLT-II, California Verbal Learning Test–Second Edition; T25FW, Timed 25-Foot Walk; MVPA, moderate-to-vigorous physical activity.

## Data Availability

The datasets presented in this article are not readily available. The raw data supporting the conclusions of this article will be made available by the authors upon reasonable request.
